# New Insights into the Biology of the Emerging Tembusu Virus

**DOI:** 10.3390/pathogens10081010

**Published:** 2021-08-10

**Authors:** Rodolphe Hamel, Thipruethai Phanitchat, Sineewanlaya Wichit, Ronald Enrique Morales Vargas, Jiraporn Jaroenpool, Cheikh Tidiane Diagne, Julien Pompon, Dorothée Missé

**Affiliations:** 1 MIVEGEC, Univ Montpellier, IRD, CNRS, 34000 Montpellier, France; c.diagne@icloud.com (C.T.D.); julien.pompon@ird.fr (J.P.); dorothee.misse@ird.fr (D.M.); 2 Department of Medical Entomology, Faculty of Tropical Medicine, Mahidol University, Bangkok 10400, Thailand; thipruethai.pha@mahidol.ac.th (T.P.); ronald.mor@mahidol.ac.th (R.E.M.V.); 3 Department of Clinical Microbiology and Applied Technology, Faculty of Medical Technology, Mahidol University, Nakhon Pathom 73170, Thailand; sineewanlaya.wic@mahidol.ac.th; 4 Medical Technology Department, School of Allied Health Sciences, Walailak University, Nakhon Si Thammarat 80160, Thailand; jjirapor@mail.wu.ac.th

**Keywords:** TMUV, emergent arboviruses, zoonosis, host-pathogen interactions, vector

## Abstract

Reported for the first time in 1955 in Malaysia, Tembusu virus (TMUV) remained, for a long time, in the shadow of flaviviruses with human health importance such as dengue virus or Japanese encephalitis virus. However, since 2010 and the first large epidemic in duck farms in China, the threat of its emergence on a large scale in Asia or even its spillover into the human population is becoming more and more significant. This review aims to report current knowledge on TMUV from viral particle organization to the development of specific vaccines and therapeutics, with a particular focus on host-virus interactions.

## 1. Introduction

Tembusu virus (TMUV) is an emerging arbovirus (arthropod-borne virus), which was initially identified in Malaysia in 1955 from *Culex tritaeniorhynchus* mosquitoes and then occasionally reported in different surveys in Southeast Asia (SEA) during the 1970s [1,2]. As with the Japanese encephalitis (JEV) and dengue (DENV) viruses, two of the most important endemic arboviruses in SEA, TMUV belongs to the genus Flavivirus in the Flaviviridae family. Until the first major outbreak of TMUV in ducks in 2010, the virus was largely neglected, and studies were scarce. Since 2000, new variants coined Sitiawan virus, duck Tembusu virus (DTMUV) or Baiyangdian virus (BYD) have been identified and reported to cause avian outbreaks [3,4,5]. However, the latter viruses are not isolated species since they are closely phylogenetically related to the original TMUV strain of 1955. Sitiawan virus, DYMUV and BYD are now classified in the TMUV clade/monophyletic group and distributed in different sub-clusters. In this review, we will refer to all these viruses as TMUV, except when specific descriptions have been associated with one sub-cluster.

TMUV causes encephalitis and neurological disorders in avian populations, resulting in morbidity rates of up to 90%, associated with damage to the female reproductive system leading to severe loss of egg production in farms [4]. Since 2010, TMUV has caused several outbreaks and has been detected repeatedly in China and SEA, underscoring the emergence and circulation of this virus outside of its original geographic region. Given the recent regional expansion and associated large economic losses in the poultry industry, TMUV should now be considered an emerging infectious disease.

To date, knowledge on TMUV ecology and biology remains incomplete, and the driving cause of its sporadic emergence remains unknown. This strongly contrasts with other emerging arboviruses causing human diseases such as yellow fever (YFV), DENV, West Nile (WNV), JEV and Chikungunya (CHIKV) viruses. In the last decade, important progress in the understanding of TMUV biology has been made through both experimental animal models and in vitro studies. Here, we review the epidemiology, virus-host interaction and cell biology of avian TMUV infection. We also discuss approaches to increase the knowledge of this virus in order to properly evaluate the associated risk of spillover in humans.

## 2. Tembusu Virus

### 2.1. Genomic Organization and Replication

**Figure 1:**
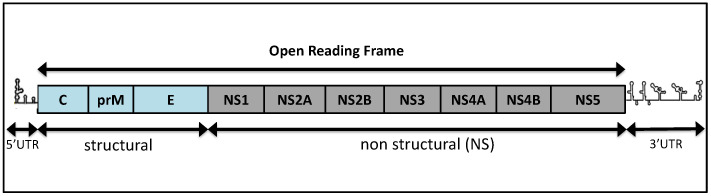
Structure of TMUV genome. The RNA genome is similar to other flaviviruses, with an open reading frame encoding three structural proteins, C, prM and E, and five nonstructural proteins NS1 to NS5. The structural and nonstructural proteins are generated after proteolytic cleavage of the polyprotein. Untranslated regions are shown schematically.

As with other flaviviruses, viruses from the TMUV group have a ~11 kb positive strand RNA genome, which is composed of a single open reading frame (ORF) flanked by 5’terminal and 3’terminal non-coding regions (UTR) (Figure 1). The 5’ end is capped with an m7GppAmp structure (type 1 cap), and the 3’ end is free from a poly A tail. The ORF is predicted to be translated into a single polyprotein that is subsequently cleaved by host and viral proteases in three structural proteins: envelope (E), membrane precursor (prM) and capsid (C), as well as seven nonstructural proteins (NS1, NS2A, NS2B, NS3, NS4A, NS4B and NS5). Viral proteins are necessary for viral replication and assembly [6]. The virion has an icosahedral capsid enclosed by a lipid envelope with a diameter of 30 to 60 nm [4,5,7].

Despite a lack of information on TMUV replication, this process is thought to be more or less identical to that of other members of the Flavivirus genus [6]. Prior to cell internalization, flaviviruses bind to a set of the cellular surface factor via cellular receptors. The recognition and internalization process involves different types of cell surface receptors and interaction with external viral proteins such as E and M proteins [8]. The E protein is involved in surface receptor binding and facilitates viral entry and the subsequent fusion steps between the viral envelope and the intracellular membranes [9]. At the cell surface membrane, the heparan sulfate receptor family, C-type lectin receptors or phosphatidylserine receptors facilitate flavivirus entry, although their exact role remains poorly understood [8]. Given its recent identification, TMUV cellular receptors are less known than for other members of the Flavivirus genus. Experiments with mammalian (BHK-21) and avian (DEF) cells using different drug treatments targeting surface receptors revealed the implication of the heparan sulfate molecule in recognition and cell attachment [10]. This precursor study has paved the way to a better understanding of TMUV attachment and entry mechanisms.

A range of endocytic pathways are usually used by flaviviruses to penetrate into the cell [11]. Zhang et al., using the hamster kidney BHK-21 cell line, reported the involvement of three different endocytosis pathways in the entry process of TMUV. The authors showed an impact on virus infection when they antagonized clathrin-dependent endocytosis by chlorpromazine and dynasore treatment or after depletion of cholesterol, suggesting involvement of clathrin- and cholesterol-dependent endocytosis in viral replication. In contrast, treatment of cells using siRNA targeting CAV1 or the genistein inhibitor shows that the caveolin pathway is not required for TMUV entry [12]. These results confirmed the first observations reported by Baloch et al. on the role of the clathrin endocytosis pathway in TMUV entry [13]. In this study, using chemical treatment and knockdown experiments, the authors also showed the involvement of the proteasome and low-pH endosome in the internalization process of TMUV. Indeed, the ubiquitin-proteasome pathway represents an important cellular proteolytic mechanism mediating a range of cellular processes including virus entry. In this respect, TMUV was reported to take advantage of this mechanism for cell entry [13]. 

To complete its cellular cycle, TMUV has to (1) translate its genomic RNA into viral proteins, (2) replicate its viral RNA genome, (3) encapsidate its genome, (4) assemble the genome into an immature virion in the endoplasmic reticulum, (5) mature the virus particles in the Golgi apparatus and (6) be secreted as infective virus particles in the extracellular space by exocytosis. Although these steps are not well known for TMUV, they do not vary among flaviviruses and have been extensively described elsewhere [14,15]. TMUV translation and replication occur in the endoplasmic reticulum (ER). Different cellular mechanisms might likely facilitate the replication, evasion and propagation of the virus, and notwithstanding recent progress [16], the exact mechanisms remain to be determined. Moreover, given the importance of the UTR regions in flavivirus replication, a comprehensive analysis of the 5′ and 3′ untranslated regions and their functions in the TMUV cycle needs to be elucidated as well. Together, the identification and characterization of host factors interacting with viral RNA and proteins are crucial for a better understanding of TMUV replication. 

TMUV presents a typical flavivirus structured 3’UTR, which plays an important role in the virus’s fitness [17]. During replication, this region leads to the formation of an RNA molecule called subgenomic flavivirus RNA (sfRNA). This highly structured RNA fragment has a critical role in viral replication and pathogenesis [18]. Thus, despite the current lack of direct evidence, sfRNA from TMUV is highly likely to have an effect on TMUV replication and pathogenesis in vertebrate hosts and vectors. It is also possible that sfRNA evolution drives modification in the viral pathogenesis pattern and thus the host range [19].

### 2.2. Phylogeny

The TMUV members belong to the *Flaviviridae* family, genus *Flavivirus* [9]. Based on the vector type, the Flavivirus genus is divided into four different groups: (1) mosquito-borne, (2) tick-borne, (3) insect-specific and (4) unknown vector flaviviruses [20]. Mosquito-borne flaviviruses (MBF) can be subdivided into *Aedes-* and *Culex*-transmitted viruses. *Aedes*-borne viruses, including DENV, YFV and Zika virus (ZIKV), are generally associated with hemorrhagic fevers and non-human primate ancestors, and those transmitted by the *Culex* genus are associated with meningoencephalitis diseases and bird reservoirs such as JEV, WNV and TMUV [21]. *Culex*-transmitted MBF are classified depending on the serology in two groups, the Japanese encephalitis serocomplex including JEV and WNV, and the Ntaya serocomplex including Ntaya virus and TMUV.

**Figure 2:**
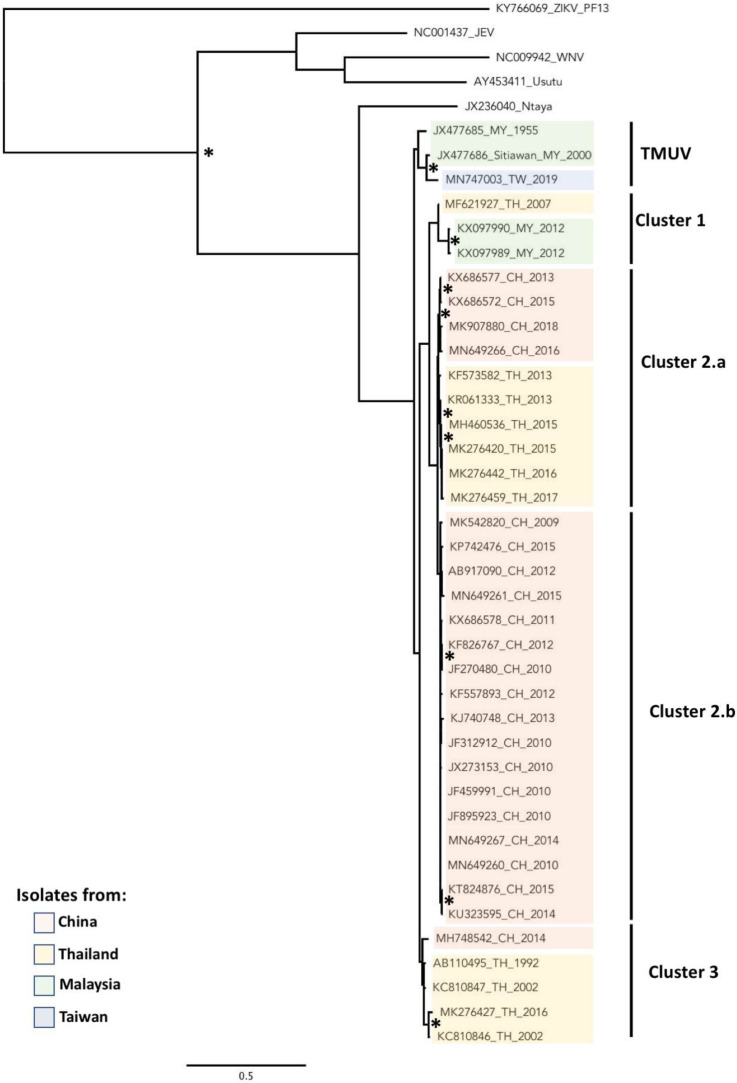
Phylogenetic analysis of TMUV strains. Non-concatenated nucleic acid sequences of the partial E genes from sequences available in the GenBank database were aligned using MEGA7. Sites that could not be unambiguously aligned were excluded, and divergent regions were excluded from subsequent analyses. Phylogenetic trees were generated using the GTR+G substitution model using PhyML software [29] and edited with FigTree v1.4.4 software (2006–2018, Source code available from: http://github.com/rambaut/figtree/). The bootstrap method was used to measure the robustness of nodes with 1000 iterations. * represents bootstrap values with a score higher than 80%. Identified TMUV strains are differentially colored in the tree depending on country origin. Annotations on the right denote TMUV cluster. All sequence accession numbers used in this review are shown in Supplementary Table S1.

The TMUV group is genetically distinct within the Ntaya serocomplex and includes homologous strains that were initially considered as single species, including Sitiawan virus [5], BYD virus [4], Perak virus [3], duck egg drop syndrome virus (DEDSV) [4,22] and duck Tembusu virus (DTMUV) [23]. These viruses are now phylogenetically linked in different sub-clusters within the TMUV group (Figure 2). The first TMUV was isolated from *Cx. tritaeniorhynchus* in Malaysia in 1955 [24] and represents the TMUV strain that is the closest to other members of the Ntaya serogroup. Virus strains related to the TMUV group were then reported in the 1990s in mosquitoes (refer to dedicated section below) in the north of Kuala Lumpur, in Sitiawan and in Sarawak, on Kalimantan island (Borneo) in Malaysia and in Thailand [1,2,25]. From our phylogenetic analysis with the available sequences, all strains isolated until 2000, except one identified in Thailand in 1992, are grouped in the TMUV cluster and are closely related to viruses belonging to the Ntaya serogroup. Interestingly, a recent strain isolated in Taiwan from *Cx. annulus* and *Cx. tritaeniorhynchus* mosquitoes was grouped in the cluster TMUV, despite the fact that no TMUV has ever been reported in Taiwan [26]. Long-distance travel via migratory birds followed by a low-level maintenance in wild birds in Taiwan may explain the recent identification of TMUV in Taiwan [27,28]. A similar spreading mode was observed for other flaviviruses such as JEV and avian influenza viruses.

Since the beginning of the 21st century, TMUV circulation has been reported in SEA and China [30]. However, these viral isolates were found to be phylogenetically separated from the TMUV cluster and have been grouped in three different lineages comprising strains either isolated in Malaysia, in Thailand or in China. A virus isolated in 2007 in Thailand formed a monophyletic lineage with two strains isolated in Malaysia in 2012 and was named Cluster 1 in our phylogenetic tree (Figure 2) [3]. In 2010, for the first time, TMUV was reported in China and caused an outbreak in egg-laying ducks [23]. TMUV was then regularly observed in China during the last decade, and a pattern of exclusive spreading in China was recently proposed [31]. Most strains isolated in China and Thailand during the last decade form a clade named Cluster 2. However, Cluster 2 is clearly subdivided into two sub-clusters, Cluster 2.a and 2.b. Interestingly, the majority of strains isolated in Thailand since 2010 and a few strains from China belong to Cluster 2.a, whereas only isolates originating from China are grouped in Cluster 2.b (Figure 2). Recently, two different research teams identified a novel cluster in the TMUV group named Cluster 3 [31,32]. Based on our phylogenetic analysis (Figure 2), we also described this Cluster 3 which gathered only viral isolates from Thailand and China. However, while Qiu et al. described only one strain from China, and Ninvilai et al. reported two strains isolated in 2014 and 2016 in China and Thailand, respectively, our analysis identified three more strains associated with this cluster that were isolated in 2002 and as early as 1992 in Thailand. This observation suggests that this new cluster is specifically located in Thailand, with a recent introduction to China. This finding is based on the tree inferred using the available E sequences.

Altogether, phylogenetic analysis suggests a circulation through insular and land territories of SEA up to the 2000s. Since 2010, TMUV has been circulating actively in China and in SEA, with potential different introductions in China from SEA. The isolation in Taiwan of a strain that groups with the original TMUV cluster has led to speculation that the viruses were introduced by migratory birds, and this may hold true for earlier introductions in Asia. In Cluster 2 and Cluster 3, the phylogenetic association between strains from China and Thailand, and in Cluster 1 between strains from Thailand and Malaysia, indicates a spatial segregation of the TMUV genotypes within Asia. However, despite increasing information on the regional phylodynamic of TMUV, many questions remain about its circulation, particularly about the dynamic of the virus in other Asian countries. Regarding the importance of regional exchange for farming and agriculture trading in Asia, and also with regard to the migration flows of birds, TMUV can be expected to continue its expansion, and this should be investigated.

## 3. Epidemiology

### 3.1. Vector

TMVU was first isolated from a *Culex tritaeniorhynchus* mosquito captured in Kuala Lumpur, Malaysia, in 1955 and has subsequently been described in different *Culex* species (Table 1). In nature, *Culex* mosquitoes appear to be the preferential vectors of TMUV as with other bird flaviviruses such as WNV and JEV. It is presently unknown whether *Aedes* mosquitoes, which are the major vectors of important flaviviruses such as DENV, are also able to transmit TMUV, although a competence study showed that *Aedes albopictus* can be infected by TMUV [33]. Recently, *Culex tritaeniorhyncus*, *Cx. quinquesfasciatus* and *Cx. pallens* were shown to be susceptible to TMUV strains isolated in China. However, despite the presence of the virus in the salivary glands of *Cx. pallens*, Guo et al. found that only *Cx. tritaeniorhynchus* and *Cx. quinquesfasciatus* were able to transmit the virus to ducks [33]. Despite the latter report and evidence for TMUV infection in *Cx. quinquefasciatus* under natural conditions [34], a recent laboratory study did not confirm the vector competence of this species for TMUV, contrary to *Cx. tritaeniorhynchus* that may be considered as a major vector. In the same line of thought, *Cx. tritaeniorhynchus* is mainly found in farms and plays a major role in the dissemination of TMUV in the bird population in Thailand [35,36]. The widespread detection of TMUV in various *Culex* species in different countries indicates that a more thorough evaluation of the vector capacities of local *Culex* species should be undertaken. This will allow authorities to assess the capacity of TMUV to spread in areas where *Cx. tritaeniorhynchus* is absent, or present in small numbers, and where other *Culex* species are endemic. The intrinsic incubation period during which the virus replicates in the mosquito to reach the salivary glands should also be evaluated to estimate the transmission capacity.

### 3.2. Vertebrate Host

The first presence of TMUV in a vertebrate host was reported in 2000 in chicks in Malaysia [5]. The TMUV host range was then reported to be similar to avian hosts of the avian influenza virus, which has caused disease in poultry in over 50 countries worldwide since 1997 [41]. TMUV was mostly isolated from ducks in industrial farms. However, the presence of the virus in free-grazing ducks has been reported in Thailand and may play an important role in the spreading of the virus through avian fauna [42]. Besides ducks, a wide range of birds including geese, chickens, sparrows and pigeons are naturally infected by TMUV (Table 2). These wild birds are likely to play a crucial role in the spread of the virus to farm birds, not only via arthropod vectors but also as a result of close contact or expectoration between animals. 

Several members of the flavivirus family, such as DENV and ZIKV, induce pathology in humans [43,44]. However, the presence of TMUV in humans or other vertebrates is not well documented. TMUV infections in humans and non-human primates were mainly detected by post-infection serological surveys, and the pathogenesis in mammals remains unclear [45]. Finally, it is not yet known whether vertebrates other than birds can serve as reservoirs or amplifying hosts, or whether they are potentiel dead-end hosts with viral loads insufficient for active transmission to mosquito vectors. Moreover, as for other flaviviruses, a high percentage of TMUV-infected individuals may be asymptomatic or present with only weak symptoms, thereby hiding the spread of the virus in the human population and the risk for a potentially large outbreak.

### 3.3. Geographic Distribution

**Figure 3:**
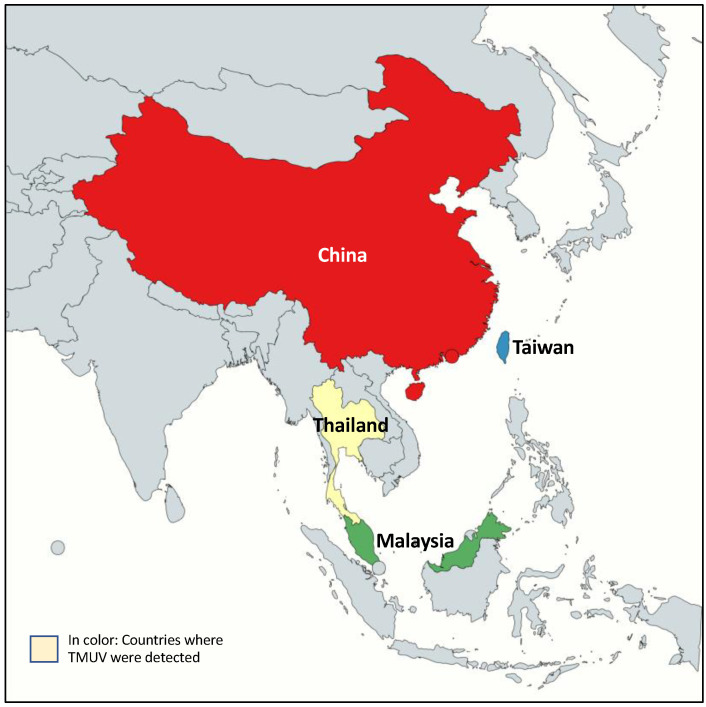
Geographic distribution of TMUV in Asia. Map representing countries where TMUV has been reported. Created with mapchart.net.

Following the first isolation of TMUV in mosquitoes in Malaysia in the mid-1950s, TMUV has been sporadically reported in SEA, mainly in wild and domestic birds and in trapped mosquitoes [2,5,25] (Figure 3). Subsequent entomological, serological and virus isolation studies pointed out a geographical distribution restricted to a few countries. While TMUV was discovered more than 70 years ago, the presence of the virus in human was not reported before 2000 with the finding of TMUV in natives and migrants in Borneo, Indonesia, and in farm workers in China [45,46]. The first marked TMUV outbreak was reported in 2010 in China in egg-laying ducks [23]. The dramatic decrease in egg production (egg drop syndrome), associated with neurological manifestations in ducks, reaching up to 90% of animals, highlighted the potential nuisance to intensive duck farming. Since then, several epizooties were reported in China, Thailand, Malaysia and Taiwan [3,26,63]. Following the identification of new TMUV variants in Thailand in 2015, a retrospective study confirmed the presence of the virus as early as 2007 [30].

Despite the lack of information about the virus distribution and its mode of transmission, human population movements and regional trade may provide a partial explanation for the distribution. The impact of bird migration may also explain the emergence of the virus in countries distant from its site of origin. Finally, as TMUV mainly affects birds, its presence may have been unreported or underestimated until farms with large numbers of fowls in close proximity were affected [46,73].

### 3.4. Transmission

**Figure 4:**
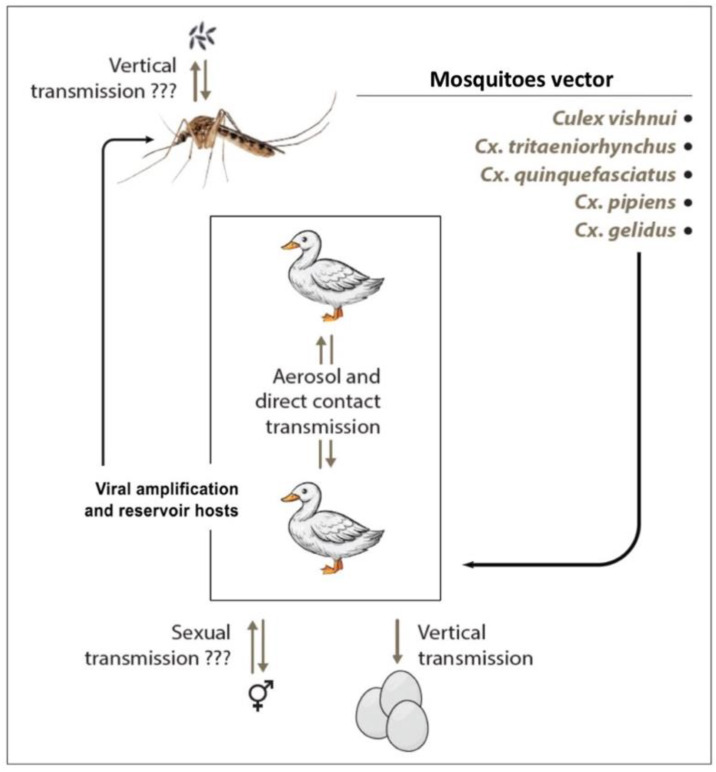
Summary of the transmission cycle for TMUV.

TMUV transmission occurs during mosquito biting on a vertebrate host (Figure 4). However, although the bite of an infected mosquito is probably the main mode of transmission, the existence of other modes may exist. Vertical transmission within mosquitoes was examined for *Cx. tritaeniorhynchus* and *Cx. Quinquefasciatus*, but the progeny was not infected [35]. Nevertheless, further studies are needed to clearly assess the vertical maintenance of infection in mosquitoes.

Birds are the main hosts of the virus, and vectors of the *Culex* genus associated with birds play a major role in transmission. Observation of TMUV-affected animals shows that the virus has been detected in the ovaries of birds and in eggs. Intrauterine transmission from mother to chick is therefore possible and leads to abortion of the gestating eggs. This clinical presentation is reminiscent of the ability of ZIKV to be transmitted in humans from mother to child during pregnancy, resulting in abortion or congenital malformations [74]. Although sexual transmission has not yet been demonstrated, non-vector transmissions were reported in birds by airborne contamination or by close contact between animals [72]. 

Maintenance of TMUV was observed in duck farms in China during the winter season when mosquitoes are inactive. To confirm non-vector transmission, naïve ducks were exposed to direct contact with infected animals or exposed to infected groups by airborne transmission. Exposed ducks developed specific acute signs of infection with increased viremia in serum and seroconversion [75]. In 2020, Ninvilai et al. evaluated the effect of duck age on TMUV infection and contact transmission. Overall, TMUV was transmitted from all age groups of infected animals to contact-naive ducks. However, naïve groups in contact with older infected ducks had higher levels of the virus in the oropharyngeal and cloacal swabs than those exposed to younger infected ducks [76].

Therefore, the regular presence of wild birds on farms, especially outdoors, could allow transmission between domestic birds and maintenance of the virus on farms without the intervention of an arthropod vector. This original mode of transmission for an arbovirus needs to be evaluated in depth because it could facilitate the spread of the virus in a region without a vector. Finally, the transmission of TMUV to non-human primates and humans has been demonstrated by serological studies, as well as by the presence of viral RNA [45,46]. The role of vertebrate hosts other than birds in viral transmission is not known at present but should be evaluated.

## 4. Clinical Features and Pathogenicity

### 4.1. Symptomatology/Disease Presentations

TMUV is a mosquito-borne epornitic flavivirus, as with WNV and Usutu virus (USUV), with its natural maintenance cycle involving birds and mosquitoes [6]. The etiology of the infection in birds is characterized by sudden acute signs including rhinorrhea, diarrhea, anorexia and perturbation of social behavior. Infected animals also show severe neurological disorders including encephalitis, difficulty to move or imbalanced movement, ataxia and paralysis [30]. The infection rate can reach 90% in a farm, depending on husbandry conditions, whereas the mortality rate is variable, ranging from 5% to 30% of the infected flocks, and is increased by secondary infections [23]. The spleen, liver, kidney and brain usually present gross lesions. Histologic analysis shows multiple microscopic changes in infected organs with hemorrhage, inflammation, hyperplasia and macrophage and lymphocyte infiltration [3,63]. The common clinical presentation of the disease observed in infected female ducks is a generalized damage to the ovaries with large inflammation, hemorrhage and hyperemia associated with organ degeneration [4,30,77]. TMUV infection is associated with a degeneration of embryos and a dramatic decrease in egg production coined “egg drop syndrome”, leading to important economic losses both in traditional or industrial poultry production in Asia [54]. A recent study reported that ducks of all ages are potentially susceptible to infection with TMUV—Cluster 2. Although a greater severity was reported in younger animals in some duck populations [78], a higher susceptibility to the infection was reported in old breeding ducks [77]. Moreover, older infected ducks present a longer shedding period with a high viral load, without clinical signs, suggesting the potential role of these animals in the spread of the virus during outbreaks [76].

It is now clearly established that TMUV is a neurotropic virus for birds, although the mechanisms of diffusion at the neuronal level are still poorly understood. However, a recent study on a duckling model shed light on the modalities of passage from the blood-brain barrier (BBB) to the central nervous system [79]. Thus, in the early stages, there are few clinical symptoms, and, although there is a propagation of the virus in the brain with a corollary induction of inflammatory cytokines, the BBB seems to remain relatively impermeable to the virus. It is therefore necessary to wait for a more advanced stage of the infection to see the first neurological symptoms associated with a disruption of the BBB and a diffusion in the microvascular endothelial cells causing an inflammatory storm and fatal encephalitis in the infected animal.

While symptoms due to TMUV infection are remarkably well described in birds, disease presentations in other vertebrate hosts are sparse. In 2020, Yurayart et al. reported neuropathogenesis and global dissemination in mice intracerebrally infected with TMUV. The animals exhibited a wide range of clinical signs with additional severe internal organ lesions that led to death [80]. Tang et al. investigated the presence of TMUV infection in avian farm workers [46]. Oral swab and serum samples were collected for molecular and serological screening for the presence of TMUV. Semi-nested RT-PCR was used as a molecular method, whereas IgG ELISA and a virus neutralization test were performed for serological assays. More than 70% of serum samples contained detectable levels of anti-TMUV antibodies, whereas TMUV could be isolated from 48% of oral swabs. Asymptomatic presentation was observed in infected workers as well. Moreover, the authors reported, for the first time, a potential spillover of TMUV to humans and suggested considering TMUV as an emergent zoonotic pathogen, although, as with other avian flavivirus members, human spillover events may represent self-limited, dead-end cases with no further human-to-human transmission. A recent study in Thailand reported seropositivity in humans for TMUV without previous specific symptoms [73]. The study highlighted the importance of prospective surveillance and survey in the human community, particularly the population at high risk for exposure to avian fauna. Further studies are also needed to better evaluate the potential outcomes in mammals, particularly neurological presentations and their role in transmission. 

### 4.2. Detection and Diagnostics

Since the first isolation of TMUV, several techniques have been developed to study the virus or detect it for diagnostic purposes. The development of diagnostic tools was particularly motivated to prevent large outbreaks in farms and preserve the poultry industry from economic losses. Prevention of outbreaks in countries producing and exporting a large amount of poultry is a major issue, especially for China as it was the first producer of ducks in the world. 

Kono et al. identified viral particles by electron microscopy in the transformed chicken B lymphocyte cell line BK3 (LSCC-BK3) infected with TMUV [5]. Although virus isolation and electron microscopy remain the gold standard, they are not suitable for diagnostic purposes. Isolation of TMUV is time-consuming, and implementation requires a dedicated laboratory with costly equipment and high-skilled trained personnel, making isolation difficult outside research laboratories. Moreover, fresh specimens must be used to obtain viable viruses. Finally, TMUV isolation from cells or embryos is mainly carried out in laboratories for basic research. 

Nowadays, common methods for diagnosis of TMUV include serological detection by enzyme-linked immunosorbent assay (ELISA), based on NS1 protein detection, and the plaque reduction neutralization test (PRNT) [81,82]. The spatial organization of the E protein has been well described in DENV, WNV and JEV, showing a high degree of similarity between flaviviruses [83,84,85]. Since this glycoprotein is exposed on the surface of the virion, it is responsible for its immunogenicity [86,87]. Indeed, the E protein has been widely used to produce mono- or poly-clonal antibodies. Recently, a new neutralizing antibody (1G2) cross-reacting with JEV, WNV and ZIKV and targeting a minimal epitope located in domain II of the E protein was reported to provide a large protection against TMUV in mice, suggesting a potential usage in detection and a valuable candidate for diagnostic and therapeutic purposes [88]. Reverse transcriptase coupled with polymerase chain reaction (RT-PCR) is commonly used to evaluate the presence of viruses in biological fluids. Several approaches using multiplex PCR targeting avian viruses were developed to detect TMUV [89,90,91]. Finally, a new and promising detection method using reverse transcriptase-PCR coupled with mass spectrometry detection has been developed to screen the presence of specific duck viruses [92]. With this technique, various viral infections of interest to duck farming can be simultaneously identified with a low detection threshold.

### 4.3. Cell Biology Tools

To ensure its transmission, TMUV must replicate in mosquito vectors prior to a vertebrate host (Figure 4). In parallel with virus isolation in embryonated chicken eggs, C6/36 mosquito cell lines were rapidly used for virus isolation [5,25]. However, the lack of information on the virus in the vertebrate host has led to the use of primary cells and cell lines to investigate the biology of the virus (Table 3).

Different types of avian cells have been used to evaluate TMUV biology and include BK3 cells, a transformed chicken B lymphocyte cell line, the chicken macrophage-derived cell line HD-11 and the DF1 cell line, isolated from chicken embryo fibroblasts. Besides specific usage for research, chicken embryo fibroblasts and duck and chicken eggs are routinely used for virus isolation and maintenance [3,4,5,26,30,93,94].

TMUV is also able to replicate in a wide range of non-avian cells which are used to assess the impact of TMUV in mammalian hosts. The primary cells and cell lines used in TMUV investigation are summarized in Table 3. Human cell lines were used to evaluate tropism of the virus and determine molecular mechanisms involved in TMUV infection. The HEK293T cell line and a mouse primary cell line (MEF) were used to investigate the innate immune response and how TMUV evades the immune response [95] (see specific paragraph). A recent investigation on TMUV cell tropism was carried out using various human cell lines. The authors pointed out the susceptibility of liver, kidney and nerve cell lines at various degrees, while the lung, muscle, B cells, T cells and monocytes were largely resistant to the infection [96]. All these reports indicate that TMUV is able to replicate in a wide range of vertebrate cells. Results suggest that targeted organs are similar in mammals and birds. However, knowledge on pathogenesis in human cells is parceled, and further investigation is needed. Furthermore, while TMUV is transmitted via mosquitoes’ bite, there is a need for more information on the biology in mosquitoes as only in vitro studies with C6/36 cells have been conducted.

### 4.4. Innate Immune Response and Viral Evasion

Flaviviruses trigger a strong innate immune response, which induces the production of a broad range of complementary antiviral molecules. Innate immunity is initiated by recognition of specific viral components, named pathogen-associated molecular patterns (PAMPs), via cellular pattern recognition receptors (PRRs) and was largely investigated in mammals and in birds [100,101,102,103]. Three classes of PRRs sense the presence of PAMPs in infected cells: Toll-like receptors (TLRs), retinoic acid-inducible gene I-like receptors (RLRs) and NOD-like receptors (NLRs). The first two are crucial for interferon (IFN) response and proinflammatory cytokine production. These molecules are both essential for eliminating viruses and for recruitment of innate and adaptive immune cells [102,104].

Several studies investigated the innate immune response against TMUV both in vivo and in vitro in avian and mammalian species. After experimental infection of female ducks or ducklings, Li et al. and Zhang et al. showed a rapid multiplication of the virus in different organs (brain, spleen, kidney, heart, pancreas, thymus or bursa of Fabricius) associated with severe lesions. The infection triggers activation of the RLR and TLR pathways and overexpression of PRRs such as RIG-I or MDA-5. This activation leads to the upregulation of interferon-stimulated genes (ISGs) from the Mx and OAS families in the brain and spleen [105,106]. Upregulation of the RLR genes RIG-I and MDA-5 induced an increase in type I IFN expression in the early phase of the infection in vivo [107]. A set of experimental infections in CEFs and in 293T cells have also shown the implication of the RLR and TLR pathways in the innate immune response to TMUV infection. Through activation of the molecular adaptors MDA5 and TLR-3, involved in the RLR and TLR signaling pathways, respectively, TMUV infection strongly increased the expression of a set of type I IFN genes and some critical ISGs (Mx1, OAS1, IFITM3 and OASL), thereby reducing virus replication [108]. Hua et al. characterized the implication of TBK1, a molecule involved in several type I IFN signaling pathways, in an experimental duck model. Using overexpression and knockdown experiments, they suggested a key role of TBK1 in the antiviral innate immune response in DEFs via IFN-ß production [109]. TBK1 signaling in mammals is initiated by DDX3 interaction with TBK1/IKKε, in order to activate ISG synthesis through IRF3/7 activation [110]. In TMUV-infected ducks, the duck-DDX3 (duDDX3) modulates the innate immune response, and duDDX3 overexpression inhibits TMUV. Nevertheless, albeit duDDX3 may influence TMUV replication, TMUV is able to inhibit duDDX3 expression, suggesting an underlying mechanism to evade the immune response [111]. 

In geese, TMUV infection triggers an immune response with an increase in proinflammatory cytokines and interferons in several organs as well as in PMBCs. Interestingly, immunohistochemical analysis presented co-localization of CD8+T cells and TMUV, associated with high cytokine expression, suggesting an activation cascade due to viral infection and leading to the establishment of an antiviral status [99]. Molecular mechanisms in specific tissues involved in the etiology of TMUV disease affecting the brain and female reproductive system were partially addressed. A global proteomic analysis of duck ovaries from infected animals revealed differential protein expression including proteins involved in cellular structure, RNA processing, innate immune response, protein biosynthesis and modification, vesicle transportation and signal transduction. Interestingly, some modulations of expression may be related to immune evasion strategies [112]. In TMUV-infected duck brains, a recent transcriptomic analysis provided information on molecular mechanisms engaged in neurovirulence and host responses. Several pathways and genes specifically related to nervous system and innate immune responses were modulated, providing evidence on the neuro-immune interactions in TMUV infection [113]. Despite efforts to shed light on the molecular mechanisms triggered by TMUV infection, intimate modalities of host responses to the infection remain incompletely known, and further studies will be necessary to uncover host-pathogen interactions.

Flaviviruses have developed different immune evasion strategies [114]. Viral proteins, particularly nonstructural (NS) proteins, antagonize crucial signaling pathways, such as virus recognition pathways and the IFN pathway, needed for an effective response [115]. Several studies also reported immune evasion mechanisms developed by TMUV. Interferon treatment of TMUV-infected avian cells has shown the absence of impact on virus replication, contrary to IFN treatment on mammalian TMUV-infected cells [95]. This observation suggests specific cellular mechanisms allowing TMUV to overcome IFN-mediated effects in avian cells specifically. Involvement of TMUV NS1 in the inhibition of RLR receptor signaling by impairment of the interaction of RIG-I and MDA5 with IPS1 was reported using reporter assay experiments. The NS1 protein interacted with the CARD domain of RLR adaptors, impeding recognition and association with virus molecule adaptors. Disruption of this interaction led to a suppression of RLR-mediated IFN-β production to finally facilitate immune evasion [98]. In 2019, Wu et al. also infected HEK293T to study the impact of the viral protein NS2B and reported an interaction with actors of the RIG-I pathway, resulting in suppression of the type I interferon response. The TMUV NS2B3 viral protease may also inhibit IFN-β production. Contrary to TMUV-NS1, the NS2B3 protease acts directly on the mitochondrial duck STING (duSTING) protein, which is a key intermediate in the RLR pathway, to inhibit signal transduction to decrease the production of IFN-β. In this context, synthesis rates of a subset of ISG-related proteins (Mx1 and OASL) are also downregulated. Interestingly, ZIKV and DENV NS2B3 present the same enzyme cleavage site as TMUV NS2B3 on duSTING [16]. In fact, ZIKV and DENV NS2B3 are able to hydrolyze duSTING, while TMUV NS2B3 is reciprocally able to cleave human STING, suggesting a potential relevance in the case of spillover of the virus to humans. A study by the same research group on the role of the NS2A protein found similar results, with an alteration in STING protein activity in RLR signal transduction. In the same way as NS2B, the binding of NS2A on STING blocks the interaction with TBK1, decreasing its phosphorylation and leading to the inhibition of IFN-β production [116]. A recent study reported that the NS4B protein can inhibit IFN-β production as previously observed with DENV. Thus, the TMUV NS4B protein has been identified as a major inhibitor of the RLR pathway decreasing the expression of RIG-I, MDA5, MAVS, STING and also TBK1, unlike NS2-A and -B. Moreover, specific mutations in the NS4B protein modifying interactions with TBK1 lead to phenotypic changes decreasing the pathogenicity of TMUV. Thus, NS4B appears to strongly interact with TBK1 and inhibit its recruitment by STING, resulting in a blockage of the signaling pathway [117]. These studies confirm the importance of TBK1 in the implementation of an efficient antiviral response, as reported by Hua et al. [109], and strategies developed by TMUV to overcome the immune response. Finally, flavivirus nonstructural protein 5 (NS5) is an RNA-dependent RNA polymerase (RdRp) that catalyzes the replication of the viral RNA genome in the replication complex [118]. Little is known about the molecular mechanisms of replication with TMUV; however, NS5 also appears to play an important role in infection. Indeed, specific amino acid substitution in the NS5 protein decreases infectivity both in vitro in BHK21 and DEF cells and in vivo in ducklings. This decrease is associated with a weaker innate immune response and a reduction in the expressions of IFN-α, -β and -γ, interleukin IL-1β and IL-6. Development of attenuated viral models stimulates new approaches to study the molecular mechanisms involved in the pathogenicity and replication of TMUV.

Autophagy is a cellular mechanism of recycling and degradation of cytoplasmic components to prevent cell death and promote homeostasis of cells exposed to an extrinsic or intrinsic stress, including viral infection [119]. Despite the host immunity defense against several pathogen infections, autophagy can be hijacked by a range of viruses, including flaviviruses such as ZIKV [120], DENV [121] or JEV [122], to promote their own replication. Induction of autophagy by TMUV was confirmed in avian cells, acting as a viral strategy to evade the host immune response [94]. In these studies, stimulation of autophagy promoted TMUV replication, and, inversely, treatment with chemical inhibitors of autophagy led to a decrease in TMUV virions. Moreover, Hu et al. showed that TMUV infection promotes degradation of the p62 autophagy adaptor, leading to a downregulation of the innate immune response mediated through the TBK1 protein and evasion of the antiviral response. Indeed, the p62 adaptor is not only a cargo adaptor but also an important player in the immune response via activation of the IFN and NF-kB pathways. p62 degradation may be a strategy developed by TMUV to evade innate immune responses [94,123].

The endoplasmic reticulum (ER) is an important intracellular organelle in the protein synthesis pathways where proteins are folded and matured before intracellular or extracellular release. The replication cycle of TMUV, as with other flaviviruses, mainly takes place in close interaction with the ER of the infected cell [124]. ER acts as the site where the viral genome is translated in the protein and replicated in the de novo genome RNA in vesicle packets, which are formed by invagination of the ER membrane. ER homeostasis is firmly regulated by a quality control process preventing accumulation of misfolded proteins in the ER lumen. However, similar to other viruses, flaviviruses trigger ER stress. To alleviate ER stress and maintain homeostasis, infected cells activate a pro-survival mechanism pathway known as the unfolded protein response (UPR). Nonetheless, flaviviruses have evolved to exploit the UPR pathway to facilitate their own replication by preventing apoptosis, promoting autophagy and evading innate immune responses [125,126]. Similarly, TMUV has been reported to activate the UPR via its three arms (IRE1, ATF6 and PERK signaling branches) both in avian and mammalian cell lines [127]. However, the exact role of the UPR pathway in TMUV replication remains unclear and needs to be further investigated.

### 4.5. Treatment and Vaccine Development

As with some other flavivirus infections, there is no specific treatment for TMUV. The antiviral efficacy of different molecules is currently being evaluated. For example, minocycline, a tetracycline analogue, has shown some efficiency in vitro against neuronal cell death infected by neurotropic viruses. This neuroprotective effect was also found with duck neuronal cells infected with TMUV [128]. Epigallocatechin-3-gallate (EGCG), a polyphenol-like active molecule, has already shown broad-spectrum activity against different viruses and appears as a promising treatment against TMUV infection. EGCG use in BHK-21 cells showed a significant decrease in TMUV replication, probably due to an increase in type I IFN production. This effect was then confirmed by an increased survival rate of TMUV-infected ducklings treated with EGCG [129]. New therapeutic strategies are being evaluated to prevent or directly treat TMUV. The use of the capsid-targeted viral inactivation (CTVI) strategy, an antiviral strategy targeting the capsid protein originally developed to target retroviruses [130] and which has shown success against DENV and JEV [131,132], has also been recently evaluated for the treatment of TMUV [133]. 

Currently, there is no commercial vaccine against TMUV. However, regarding the large spread of the virus in Asia since the first outbreak in China in 2010 and the association with a high economic impact on the poultry industry, the development of an effective vaccine is a primary objective. Since the first report of the virus, different research teams have taken on the challenge. In China, attenuated viruses by passaging in embryonated chicken eggs were developed as a live vaccine strategy [134,135]. The attenuation of virulence might be due to amino acid substitution in structural and nonstructural proteins, but the attenuated viruses keep their immunogenicity, thereby providing effective protection for animals against TMUV infection. Different types of oil emulsion-containing inactivated TMUV vaccines were developed to protect ducks against TMUV [136]. In 2017, Zhang et al. proposed using an inactivated TMUV from the TMUV-JXSP strain which was successfully propagated in the BHK-21 cell line, then inactivated using β-propiolactone and associated with medical-grade white oil to obtain an oil emulsion vaccine amenable for injection. Vaccinated ducks presented an increase in antibody titers after the first injection and experienced a diminution of viral load without apparition of the egg drop effect [137]. In the same way, after several passages of TMUV isolated from sparrows in China, attenuated viruses possessed a strong immunogenicity and provided an effective protection for ducks exposed to TMUV [138]. Recently, the team of Yang et al. worked with a variant of TMUV which developed attenuated virulence after several passages. Their work shed new light on the development of an effective vaccine against TMUV infection, determining that residue 304 of the E protein was essential for virulence and cell attachment [139]. However, despite the interest of these candidate vaccines, further investigations are needed to optimize certain aspects of vaccination such as the route of inoculation, the optimal number of inoculations to achieve the correct immunity or possible interactions with other animal vaccination programs.

Two recombinant duck enteritis viruses were also designed to express a part of the TMUV E protein and the pre-membrane protein. Immunization of ducks led to the production of protective neutralizing antibodies, and further TMUV-challenged animals exhibited resistance to the infection [140]. A reverse genetic strategy was used to produce a chimeric recombinant TMUV based on the JEV backbone, which was successfully used in mice [141]. In 2016, Ma et al. used liposomes containing a recombinant TMUV E protein produced in BL21 cells to immunize ducks. Two injections induced an effective immune response leading to full protection to ducks challenged with TMUV compared to animals challenged with Freund’s adjuvant as a control. Their study suggested that a liposome-based vaccine is an interesting candidate. The use of a combination of liposomes (for delivery) and protein E (for immunization) presents several advantages such as better delivery, higher stability, longer immunization and lower toxicity [142]. A vaccine based on the adenovirus platform was also experimented. The E protein of DTMUV expressed in a recombinant adenovirus triggered an immune response and antibody production in immunized ducks. Vaccine-challenged ducks presented an up to 80% survival rate, indicating effective protection [143].

Recently, vaccine development efforts were dedicated to providing a recombinant vaccine against TMUV. Several studies reported the development of antibodies targeting immunogenic domain regions of the virus particles, particularly the loop of domain II of the E protein [88]. Among them, a DNA-based vaccine was constructed to target viral prM-E proteins. The DNA-based vaccine provided effective immunization and opened up an interesting avenue for vaccination methods. By using attenuated Salmonella SL7207 bacteria as a vehicle to deliver the prM and E proteins, a trial showed the development of effective protection in ducks [144]. Other teams used a DNA vaccine also based on the integration of prM and E proteins but coupled with CpG oligodeoxynucleotide as an adjuvant to boost the protection efficiency and showed production of neutralizing antibodies providing protection to ducks challenged with TMUV [145]. Chimeric virus-like particles (VLPs) containing the E protein of TMUV showed their potential immunization effect in birds, leading to a decrease in the viral load in immunized birds [146].

Strategies using attenuated pathogens are widely used for vaccine development. However, an interesting way to develop new vaccines would be to use the strategy based on messenger RNA (RNA vaccine) as it is currently being utilized with success against SARS-CoV2. This type of strategy, coupled with liposome delivery, is already being used in attempts to develop a vaccine against other flaviviruses such as DENV and ZIKV [147]. Despite the extensive research on vaccines and treatment, the lack of effective means against TMUV calls for further research.

## 5. Conclusions

As with other epornitic arboviruses, TMUV has taken advantage of increasingly favorable conditions over recent decades to spread across Asia and invade new territories. The first outbreaks in China in 2010 led to a reconsideration of the danger of this virus. Its transmission is largely facilitated by the conditions of intensive poultry rearing in farms, containing several thousands of animals. In such conditions, TMUV even seems to spread between animals without the intervention of mosquito vectors. Finally, the impact of migratory birds has not been clearly demonstrated but could play a significant role in the expansion of the virus across Asia. Thus, intensive animal husbandry, increasing trade and environmental changes have facilitated the wide spread of TMUV. 

This situation is all the more worrying since the passage of this virus to humans has been demonstrated and thus could lead to the emergence of a new zoonotic disease. The clinical presentation of TMUV infections in birds is similar to that of JEV, WNV or ZIKV, and it is easy to imagine the impact on the human population if TMUV were to develop therein. Due to the lack of widespread surveillance, the virus has, thus far, been detected in only three Asian countries and actively studied only in China and Thailand. However, the remoteness of these countries and the phylogenetic pattern of the virus suggest that the virus is more widely established in Asia and SEA.

In recent years, research on host-pathogen interactions has mainly been focused on avian models, but a better understanding of the biology of the virus in mammals and, in particular, in humans is important. Finally, the presence of potential vectors in non-Asian regions may facilitate spreading outside of Asia as it has recently been the case for other arboviruses. Thus, in order to respond to the challenges posed by this emerging virus, an integrated One Health approach could allow a broader understanding of the different parameters involved in the ecology of TMUV. 

## Figures and Tables

**Table 1:** Mosquito species found infected by TMUV.

Country/Location of Origin	Species	Year	References
Kuala Lumpur, Malaysia	*Cx. tritaeniorhynchus*	1955	[24]
Malaysia	*Cx*. *vishnui*	1970	[2]
Kamphaengphet Province, Thailand	*Cx*. *gelidus* *Cx*. *tritaeniorhynchus* *Cx*. *vishnui*	1982	[25]
Chiang Mai Province, Thailand	*Cx*. *tritaeniorhynchus*	1992	[1]
Kamphaeng Phet Province, Thailand	*Cx*. *vishnui*	2005	[37]
Shandong Province, China	*Cx*. *pipiens*	2010–2012	[38]
Yunnan Province, China	*Cx*. *tritaeniorhynchus*	2012	[39]
Shandong Province, China	*Cx*. *spp*	2012	[40]
Sing Buri Province, Thailand	*Cx*. *tritaeniorhynchus*	2015	[36]
Kanchanaburi Province, Thailand	*Cx*. *quinquefasciatus*	2015	[34]
Taipei, Taiwan	*Cx. annulus*	2019	[26]
Taichung, Taiwan	*Cx. tritaeniorhynchus*	2019	[26]

**Table 2:** Vertebrate hosts found naturally infected by TMUV.

Country/Location of Origin	Species	Year	References
Perak State, Malaysia	Broiler chick	2000	[5]
Thailand	Duck	2007	[30]
Shandong Province, China	Human	2010–2012	[46]
Shanghai, China	Duck	2010	[47]
Shandong Province, China	Meat duck Layer duck	2010	[48]
Henan Province, China	Layer duck	2010	[48]
China	Pekin duck, Cherry Valley Pekin duck Shaoxing duck Breeder duck	2010	[4,23,49,50]
China	Goose	2010	[49]
China	Chicken	2010	[51]
Jiangsu Province, China	Goose	2010	[52,53]
Shandong Province, China	House sparrow *(Passer domesticus*)	2010–2011	[54]
Guangdong Province, China	Layer duck	2011	[55]
Guangxi Province, China	Layer duck	2011	[48]
Shandong Province, China	House sparrow	2012	[40]
Beijing Autonomous City, China	Pigeon	2012	[22]
Shandong Province, China	Goose	2012	[40,56]
Hebei Province, China	Duck	2012	[22]
Jiangsu Province, China	Egg-laying duck	2012	[48]
Shandong Province, China	Duck	2012	[48,56]
Fujian Province, China	Duck	2012	[57]
Malaysia	Pekin duck	2012	[3,58]
China	Duck	2013	[59]
Guangxi Province, China	Cherry Valley duck	2013	[60,61]
Shanghai Province, China	Pekin duck	2013	[62]
Shandong Province, China	Layer duck	2013	[40]
Anhui Province, China	Layer duck	2013	[40]
Thailand	Duck	2013	[63]
Thailand	Broiler chicken	2013	[32,64]
China	Chicken	2013	[40]
China	Goose	2014–2015	[65]
China	Chicken	2014	[66]
China	Layer duck	2014	[67]
China	Mallard *(Anas platyrhynchos)*	2014	[68]
China	Broiler duck	2015	[69]
China	Layer duck	2015	[70]
Guangdong Province, China	Muscovy duck	2015	[71]
Thailand	duck	2015	[72]
Shandong Province, China	Meat duck	2016	[40]
Inner Mongolia Autonomous Region, China	Meat duck	2017	[40]
Thailand	Free-grazing duck	2018	[42]

**Table 3:** Cell models used for TMUV investigations.

	Name	Organism, Tissue	Type	Reference
Avian	DF-1	Chicken, embryo fibroblast	Cell line	[3,26,93]
	HD11	Chicken, bone marrow macrophage	Cell line	[97]
	DEF	Duck embryonic fibroblast	Primary cells from 9-day-old duck embryos	[10,98]
	goose PBMCs	Goose blood	Primary cell	[99]
	LSCC-BK3	Chicken, B lymphocyte	Cell line	[5]
Mammalian	MARC145	Monkey kidney	Cell line	[5]
	VERO	African green monkey kidney	Cell line	[3,5,26,58,95]
	BHK-21	Hamster, kidney	Cell line	[10,22,26]
	CPK	Porcine, kidney	Cell line	[5]
	MEF	Mouse embryonic fibroblast	Primary cells	[98]
	A549	Human, epithelial lung	Cell line	[95,96]
	HeLa	Human, epithelial cervix	Cell line	[95]
	SH-SY5Y	Human, epithelial bone marrow	Cell line	[95,96]
	HEK293T	Human, epithelial kidney	Cell line	[96,98]
	HUH7	Human, liver	Cell line	[96,98]
	RD	Human rhabdomyosarcoma cell	Cell line	[96]
	SUP-T1	Human T cell lymphoblastic lymphoma cell	Cell line	[96]
	Z-138	Human B cell non-Hodgkin’s lymphoma cell	Cell line	[96]
	U937	Human histiocytic lymphoma cell	Cell line	[96]
	imHC	Human hepatocyte-like cell	Derived cell from hiPSC	[96]
Insect	C6/36	*Aedes albopictus*, larva	Cell line	[22,26,58]

## Supplementary Materials

The following are available online at https://www.mdpi.com/article/10.3390/pathogens10081010/s1, Table S1: Referenced virus strains used in this study.
