# Supplementary material for: New Insights into the Biology of the Emerging Tembusu Virus
Source: Pathogens. 2021 Aug 10;10(8):1010. doi: 10.3390/pathogens10081010 (PMC8398659; doi:10.3390/pathogens10081010)
Supplement: Supplementary file 1 [file pathogens-10-01010-s001.zip › pathogens-1311271-supplementary.pdf]

Table S1. Referenced virus strains used in this study

Virus	GenBank accession n°	Year	Country
Tembusu virus	JX477685	1955	Malaysia
Tembusu virus	AB110495	1992	Thailand
Tembusu virus	KC810847	2002	Thailand
Tembusu virus	KC810846	2002	Thailand
Tembusu virus	MF621927	2007	Thailand
Tembusu virus	JX273153	2010	China
Tembusu virus	JF270480	2010	China
Tembusu virus	MN649260	2010	China
Tembusu virus	JF895923	2010	China
Tembusu virus	JF312912	2010	China
Tembusu virus	JF459991	2010	China
Tembusu virus	KX686578	2011	China
Tembusu virus	KF557893	2012	China
Tembusu virus	KF826767	2012	China
Tembusu virus	KX097989	2012	Malaysia
Tembusu virus	AB917090	2012	China
Tembusu virus	KX097990	2012	Malaysia
Tembusu virus	KR061333	2013	Thailand
Tembusu virus	KJ740748	2013	China
Tembusu virus	KF573582	2013	Thailand
Tembusu virus	KX686577	2013	China
Tembusu virus	MH748542	2014	China
Tembusu virus	MN649267	2014	China
Tembusu virus	KU323595	2014	China
Tembusu virus	KP742476	2015	China
Tembusu virus	KX686572	2015	China
Tembusu virus	KT824876	2015	China
Tembusu virus	MK276420	2015	Thailand
Tembusu virus	MH460536	2015	Thailand
Tembusu virus	MK276427	2016	Thailand
Tembusu virus	MK276442	2016	Thailand
Tembusu virus	MN649266	2016	China
Tembusu virus	MK276459	2017	Thailand
Tembusu virus	MK907880	2018	China
Tembusu virus	MK542820	2019	China
Tembusu virus	MN747003	2019	Taiwan
Ntaya virus	JX236040	2013	
Sitiawan	JX477686	2000	Malaysia
ZIKV	KY766069	2013	French Polynesia
JEV	NC001437	1989	Japan
Usutu	AY453411	2001	Austria
WNV	NC009942	1999	USA
